# Health staff perceptions of patient safety and associated factors in hospitals in Vietnam

**DOI:** 10.3389/fpubh.2023.1149667

**Published:** 2023-10-26

**Authors:** Nguyen Thi Hoai Thu, Bui Thi My Anh, Nguyen Thi Thu Ha, Doan Ngoc Thuy Tien, Pham Huong Giang, Tran Thi Nga, Nguyen Hoang Nam, Phung Thanh Hung

**Affiliations:** ^1^Department of Health Management and Organization, School of Preventive Medicine and Public Health, Hanoi Medical University, Hanoi, Vietnam; ^2^Department of Health Economics, School of Preventive Medicine and Public Health, Hanoi Medical University, Hanoi, Vietnam; ^3^Institute of Development Policy, University of Antwerp, Antwerp, Belgium; ^4^Department of International Economics, Foreign Trade University, Hanoi, Vietnam

**Keywords:** patient safety, patient safety culture, hospital survey on patient safety culture (HSOPSC), hospital, health staff, Vietnam

## Abstract

**Introduction:**

Patient safety is a global challenge of preventing and mitigating medical errors which might harm patients during their course of treatment and care. This study was employed to contribute to the existing literature aimed to assess patient safety culture among health staff and to determine predictors of health staff perceptions of patient safety in hospitals in Vietnam.

**Methods:**

A cross-sectional study was conducted in three hospitals of Vietnam with a total of 763 participants. This study used the Hospital Patient Safety Scale developed by the American Health and Quality Research Organization.

**Results:**

In general, 8 of 12 patient safety dimensions in two hospital; and 10 of 12 dimensions in a third hospital had average scores of 60% and above positive responses. The communication openness and organizational learning dimensions were found to be significant different when comparing hospitals. Regarding sample characteristics, department (subclinical department) and health staff positions (nurses/technicians, pharmacists) were significant predictors in the total model including three hospitals (*R*^2^ = 0.07).

**Conclusion:**

This study reported that communication openness and organization learning are two aspects that need to be improved they are strongly related to patient safety culture and to knowledge exchange among health staff. It has been suggested that hospitals should deliver patient safety training courses and establish a supportive learning environment to improve these challenges.

## Introduction

Patient safety is a global challenge of preventing and mitigating medical errors (both active and latent errors) which might harm patients during their course of treatment and care (1). Adverse events are reported as one of the 10 global leading causes of morbidity and mortality of which approximately 50% are avoidable (2). Moreover, the most common medical errors were associated with diagnosis and medication errors (3). The patient safety culture of an organization is the outcome of individual and organization shared values, attitudes, perceptions, competencies and patterns of behavior that determine the commitment, style, and competence of managing the health and safety of an organization (4). The safety culture, an important attribute of the health system, reflects the quality of healthcare services being supplied, the level of system credibility and the resilience of adverse events (5).

Evaluating the patient safety culture of health organizations receives increased attention, especially in hospitals where patient-centered care comes before other operational targets. The Agency for Healthcare Research and Quality (AHRQ) has developed a patient safety culture assessment, namely, Hospital Survey on Patient Safety Culture (HSOPSC) (6). HSOPSC evaluates health staff perceptions of patient safety culture. This multidimensional tool has been validated in several study contexts and is widely applied in patient safety research (7–12). HSOPSC is used to reflect the present status of patient safety of a healthcare organization, identifying the strengths and weaknesses of safety culture expressed by dimensions; in turn, improving the safety culture state and quality of healthcare services in an organization.

In Vietnam, from 2013, the Ministry of Health (MoH) constructed patient safety regulations, procedures and technical guidelines for health organizations, health staff and healthcare services. Although the MoH has taken actions on encouraging patient safety in health organizations, little is known about health staff perceptions of safety culture. The General Hospital of Agriculture, Vietnam National Children’s Hospital, Hanoi Obstetrics and Gynecology Hospital are located in Hanoi which have quality management departments with one of the primary missions being to enhance patient safety culture within hospitals. Accordingly, this study was conducted employing the HSOPSC scale with two objectives: (i) to assess the patient safety culture among health staff and (ii) to determine predictors of health staff perceptions of patient safety in hospitals in Vietnam.

## Materials and methods

### Research design and location

A cross-sectional study was conducted in one general hospital and two specialized hospitals in Vietnam including Vietnam National Hospital of Pediatrics (Hos1), Hanoi Hospital of Obstetrics and Gynecology (Hos2) and General Hospital of Agriculture (Hos3).

### Sample size and participants

A total of 763 health workers working at the three hospitals were selected for interview, including 252 health workers in Hos1, 286 health workers in Hos2 and 225 health workers of Hos3.

### Measurements

In this study, we used the HSOPSC developed by the AHRQ. The tool was officially published in November 2004 and was used in many countries around the world (13). Until March of 2017, this tool was used to survey hospitals in 71 countries and was translated to 32 different languages (Vietnamese was as the 31st language) (14). The Vietnamese version of the questionnaire was first verified by Tran Nguyen Nhu Anh in 2015 (15), and used to survey in 43 hospitals in Ho Chi Minh City. It helped hospitals to understand the perceptions, attitudes and behaviors of health workers, contributing to improve the quality of medical examination and treatment in hospitals (16).

The questionnaire included 42 questions covering 12 safety culture dimensions: communication openness, feedback and communication about errors, handoffs and transitions, management support for patient safety, non-punitive response to errors, organizational learning, overall perception of patient safety, staffing, supervisor/manager expectations and actions promoting safety, teamwork across units, teamwork within units and frequency of events reported. Each dimension consisted of three or four questions, assessed on a five-point Likert scale ranging from 1 = “strongly disagree” to 5 = “strongly agree or from 1 = never to 5 = always.

The demographic characteristics included age (under, 36–50 or 50–60 years old), sex (male or female), department (Clinical, Subclinical or Administration departments), position (Doctors, Nurses/Technicians or Pharmacists), managerial level (Leaders/managers or Staff), professional experience in years (under 5 years, 5 years to 10 years or over 10 years) were measured using single items for prospective inclusion as control variables in the analysis.

### Data management and analysis

Data were analyzed using STATA 20.0. Descriptive statistics such as mean, median, standard deviation, frequency and percentage were used to describe safety culture. For items that were positively worded, responses on 4 and 5 (“agree/strongly agree” or “most of the time/always”) on a 5-point Likert scale indicated positive responses, while 1 and 2 (“strongly disagree/disagree” or “never/rarely”) indicated positive responses on negatively worded items. The positive rate of each dimension was determined by averaging the percentages of positive responses of the subsections in each field, using the Hospital Survey Excel Tool 1.72010 of Microsoft Excel provided by the AHRQ organization and Stata Software 20.0 (4, 17). The t-test was applied to assess differences among patient safety culture dimensions, overall perceptions of patient safety and incident reporting. The effect sizes of the mean differences were calculated using Eta-squared. Multiple regression analyses with overall perceptions of patient safety as outcome was conducted, referring to how many standard deviations a dependent variable will change, per standard deviation increase in the predictor variable (18).

#### Ethics consideration

The study protocol was approved by the scientific panel from the School of Preventive Medicine and Public Health, Hanoi Medical University in Vietnam. Participation of all respondents was anonymous and voluntary. They were informed about research content and objectives as well as how the interview data would be documented and reported and that their confidentiality would be respected. Participants provided verbal informed consent and could withdraw at any time.

## Results

Regarding the characteristics of study subjects, female accounted for the majority in hospitals, from 68 to 86%. The age of the most studied subjects was under 35, from 54.8 (in Hos1) to 69.3% (Hos3). Regarding working positions, nurses accounted for the highest proportion, from 60.4 (in Hos3) to 76.6% (in Hos1), followed by doctors ranging from 19.6 to 29.3%. Staff comprised 88.5 and 94.2% of the study participants (Table 1).

**Table 1:** General characteristic of participants.

Characteristic		Hos1 n (%)	Hos2 n (%)	Hos3 n (%)
Sex	Male	35 (13.9%)	56 (19.6%)	72 (32%)
	Female	217 (86.1%)	230 (80.4%)	153 (68%)
Age in years	<35	138 (54.8%)	179 (62.6%)	156 (69.3%)
	36–50	106 (42.1%)	98 (34.3%)	69 (30.7%)
	50–60	8 (3.2%)	9 (3.1%)	
Department	Clinical department	252 (100%)	137 (47.9%)	150 (66.7%)
	Subclinical department		107 (37.4%)	25 (11.1%)
	Administration departments		42 (14.7%)	50 (22.2%)
Position	Doctors	59 (23.4%)	56 (19.6%)	66 (29.3%)
	Nurses/technicians	193 (76.6%)	191 (66.8%)	136 (60.4%)
	Pharmacists		2 (0.7%)	15 (6.7%)
	Other		37 (12.9%)	8 (3.6%)
Managerial level	Leader/managers	29 (11.5%)	24 (8.4%)	13 (5.8%)
	Staff	223 (88.5%)	262 (91.6%)	212 (94.2%)
Professional experience in years	<5 years	57 (22.6%)	138 (48.3%)	80 (35.6%)
	5–10 years	78 (31%)	87 (30.4%)	73 (32.4%)
	>10 years	117 (46.4%)	61 (21.3%)	72 (32%)

### Patient safety culture among health staff in hospitals in Vietnam

Eight of 12 patient safety dimensions in Hos1 and Hos3, and 10 of 12 dimensions in Hos2 had an average score of more than 60% positive responses (Table 2). The eight dimensions with an average score of more than 60% positive responses were the same for Hos1 and Hos3. The additional two dimensions with an average score of more than 60% positive responses were communication openness and staffing. Hos2 had higher scores than Hos1 and Hos3 on all dimensions, except on management support for patient safety and overall perception of patient safety which were lower than in Hos1. In all hospitals, the lowest scores were on handoffs and transitions.

**Table 2:** Average positive response rate of 12 dimensions.

No	Patient safety culture dimension	Hos1	Hos2	Hos3
1	Communication openness	35.3	64.7	51.6
2	Feedback and communication about errors	81	75.2	74.2
3	Handoffs and transitions	19.8	17.8	25.3
4	Management support for patient safety	78.2	77.6	72.9
5	Nonpunitive response to errors	88.1	89.2	81.8
6	Organizational learning	85.3	86.7	79.6
7	Overall perception of patient safety	97.6	93.4	85.8
8	Staffing	36.1	81.5	42.7
9	Supervisor/manager expectations and actions promoting safety	62.7	72.4	69.3
10	Teamwork across units	89.3	97.6	87.1
11	Teamwork within units	45.6	52.8	44.4
12	Frequency of events reported	73.8	80.1	68
	**Overall patient safety grade**	**27.4**	**52.4**	**35.6**

Results from *t*-tests showed significant differences in communication openness, feedback and communication about errors, management support for patient safety, organizational learning, staffing, teamwork across units, teamwork within units, and frequency of events reported between Hos1 and Hos2 (Table 3). The size of the mean differences was moderate, ranging from −0.1 to −0.28, except for staffing (MD = −0.49). Between Hos1 and Hos3, the differences were in communication openness, no-punitive response to errors, organizational learning, overall perception of patient safety, staffing and frequency of events reported. The size of the mean differences was moderate, ranging from 0.09 to 0.11, except for staffing (MD = 0.45). Only significant differences in were observed communication openness (MD = −0.19), organizational learning (MD = −0.14) and teamwork across units (MD = −0.2) between Hos1 and Hos3.

**Table 3:** Average score of 12 dimensions among Hos1, Hos2, and Hos3.

No	Patient safety culture dimension	Hos1		Hos2		Hos3		MD, *t*, *p* (Hos1–Hos2) M	MD, *t*, *p* (Hos2–Hos3) SD	MD, *t*, *p* (Hos1–Hos3) M
		*M*	*SD*	*M*	*SD*	*M*	*SD*			
1	Communication openness	3.73	0.41	4.01	0.48	3.92	0.48	**−0.28, −7.22, 0**	**0.09, 2.11, 0.035**	**−0.19, −4.52, 0**
2	Feedback and communication about errors	4.08	0.5	4.18	0.59	4.15	0.66	**−0.1, −2.16, 0.031**	0.03, 0.61, 0.543	−0.07, −1.23, 0.218
3	Handoffs and transitions	2.5	1.21	2.55	1.28	2.4	1.47	−0.05, −0.51, 0.61	0.16, 1.29, 0.199	0.1, 0.84, 0.401
4	Management support for patient safety	3.99	0.46	4.09	0.6	3.99	0.6	**−0.1, −2.08, 0.038**	0.1, 1.93, 0.054	0.01, 0.17, 0.863
5	Non-punitive response to errors	4.27	0.42	4.3	0.55	4.19	0.56	−0.03, −0.78, 0.436	**0.11, 2.27, 0.024**	0.08, 1.74, 0.083
6	Organizational learning	4.09	0.46	4.34	0.54	4.24	0.51	**−0.24, −5.61, 0**	**0.1, 2.13, 0.034**	**−0.14, −3.18, 0.002**
7	Overall perception of patient safety	4.25	0.41	4.29	0.61	4.19	0.66	−0.04, −0.9, 0.369	**0.1, 1.75, 0.081**	0.06, 1.17, 0.242
8	Staffing	3.51	0.58	4.01	0.46	3.56	0.8	**−0.49, −10.76, 0**	**0.45, 7.43, 0**	−0.05, −0.73, 0.469
9	Supervisor/manager expectations and actions promoting safety	3.85	0.53	3.94	0.79	3.9	0.73	−0.09, −1.52, 0.129	0.03, 0.47, 0.638	−0.06, −0.94, 0.349
10	Teamwork across units	4.25	0.6	4.48	0.48	4.45	0.61	**−0.22, −4.73, 0**	0.02, 0.43, 0.669	**−0.2, −3.64, 0**
11	Teamwork within units	3.51	0.75	3.65	0.71	3.51	0.85	**−0.14, −2.26, 0.024**	0.14, 1.95, 0.052	0, −0.06, 0.95
12	Frequency of events reported	3.99	0.48	4.14	0.5	4.02	0.67	**−0.15, −3.46, 0.001**	**0.11, 2.1, 0.036**	−0.03, −0.63, 0.53
	**Overall patient safety grade**	3.85	0.32	4.02	0.34	3.89	0.37	−0.17, −5.9, 0	0.12, 3.86, 0	−0.04, −1.4, 0.164

Bold text indicate statistically significant at 5% level.

### Predictors of health staff perceptions of patient safety in hospitals in Vietnam

In the multiple regression analyses of overall patient safety, with sample characteristics as predictors, department (subclinical department) and position (nurses/technicians, pharmacists) were significant predictors in the total model including all three hospitals (*R*^2^ = 0.07) (Table 4). The total explained variance was higher in Hos2 (*R*^2^ = 0.16) than in Hos3 (*R*^2^ = 0.08) and in Hos1 (*R*^2^ = 0.02). In Hos2, pharmacists was the strongest predictor for overall patient safety, followed by age in years 50–60 and managerial level staff; the least important predictor was Male sex. In Hos3, the only significant predictor was Pharmacists, while no significant predictor was noted in Hos1.

**Table 4:** Multiple regression analysis of overall patient safety.

No	Characteristic		Hos1 (*n* = 252)			Hos2 (*n* = 286)			Hos3 (*n* = 225)			Total (*n* = 763)		
			*B*	*SE*	*β*	*B*	*SE*	*β*	*B*	*SE*	*β*	*B*	*SE*	*β*
1	Sex	Male	−0.02	0.067	−0.056	0.089	0.052	**0.256**	0.066	0.054	0.188	0.044	0.032	0.125
2	Age in years	36–50	−0.03	0.055	−0.086	0.049	0.047	0.14	0.016	0.069	0.046	0.028	0.033	0.079
		50–60	−0.073	0.126	−0.208	0.256	0.121	**0.732**	–	–	**–**	0.107	0.089	0.307
4	Department	Clinical department	–	–	–	−0.082	0.066	−0.235	0.044	0.063	0.125	−0.038	0.041	−0.108
		Subclinical department	–	–	–	0.081	0.073	0.233	0.118	0.094	0.338	0.158	0.048	**0.451**
5	Position	Nurses/technicians	−0.062	0.054	−0.178	−0.121	0.051	**−0.347**	−0.096	0.058	−0.274	−0.103	0.031	**−0.296**
		Pharmacists	–	–	–	0.542	0.232	**1.551**	−0.367	0.107	**−1.051**	−0.245	0.087	**−0.701**
		Other	–	–	–	−0.119	0.077	−0.34	−0.166	0.149	−0.476	−0.066	0.061	−0.188
6	Managerial level	Staff	−0.11	0.070	−0.316	0.166	0.078	**0.475**	−0.069	0.109	−0.199	0.004	0.047	0.01
7	Professional experience in years	5–10 years	−0.027	0.056	0.07	0.045	0.2	0.07	−0.056	0.061	−0.159	0.011	0.031	0.031
		>10 years	−0.037	0.063	0.083	0.060	0.237	0.083	−0.045	0.079	−0.129	−0.004	0.038	−0.012
		*R* ^2^			0.02			0.16			0.08			0.07

Bold text indicate statistically significant at 5% level.

## Discussion

This study was the first of its snapshot that captured the perception of healthcare staff regarding patient safety culture in three different hospitals in Vietnam. To reveal all the health staff in those hospitals we applied an HSOPSC questionnaire to measure the perception on patient safety culture among health staff (17). According to the findings, the overall score for all the 12 dimensions of patient safety culture among health staff in three different hospitals ranged from 27.4 to 52.4%, which was lower than the AHRQ 2018 benchmark report of 65% or other studies in India, the US, Norway or Netherlands (ranging from 22 to 87%) (19–23). Such a difference in the average score of patient safety culture might have been due to the characteristics of hospitals, i.e., public hospital or specialized hospital and categories of health staff in each study. However, this study observed that the overall perception of patient safety in three hospitals rated at 85.8–97.6%. This finding is higher that the values reported in related studies (21–23).

Among the patient safety dimensions, this study indicated that teamwork across units held the highest positive composite score (87.1–97.6%) which reflected that health staff in three research hospitals supported and treated their colleagues with respect and a congenial working atmosphere was noted across the hospital’s units. This observation was consistent with the findings in related hospitals, indicating that teamwork across units was one of highly rated dimensions of patient safety culture. Additionally, function of good teamwork was revealed as an essential factor when steering patient safety improvements (20). Regarding of dimensions such as feedback and communication about errors “and frequency of events reported “in three hospitals positive responses ranged from 74.2 to 81.0% and from 68.0 to 80.1%, respectively. These findings implied that those hospitals in Vietnam could create an atmosphere where reporting errors was without fear or penalty to enable patient safety culture in their hospitals. These observed scores were higher than the measurements reported in other studies (19–23). Subsequently, this result showed that other patient safety dimensions such as organizational learning” reached a high positive response from 79.6 to 86.7% and “Nonpunitive response to errors” from 81.8 to 89.2%. This findings was consistent with other studies indicating that those hospitals inspired their health workforces to learn from their errors and consider their health staff as key players to improve patient safety culture (4, 19).

Concerning other dimensions of patient safety culture, this study found the dimensions with a lower positive response included “handoffs and transitions” (from 17.8 to 25.3%), especially in Hos.1 and Hos.3, “staffing” (36.1–42.7%) and “communication openness” (35.3–51.6%). Those were identified as weak areas requiring patient safety improvement. The observed differences among the hospitals and research in identifying weak areas of patient safety culture dimensions might have caused the nature or characteristics of hospital settings. These findings demonstrated that sharing patients’ data while exchanging duties with health staff was not conducted properly. Moreover, the fear of health staff regarding making mistakes in communication could have affected the patient safety performance among health staff (20–23).

In our study, communication openness and organizational learning were found to significantly differ among hospitals. Several studies also showed that the perceptions of health workers on some aspects of the patient safety culture vary widely even within the same country, only in different workplaces (20). Communication openness regarding patient safety is influenced by the organizational culture, in the way of leadership and management style (24). This problem needs to be solved because communication openness is a necessity that helps to improve health worker’s expertise, and enhances teamwork among members, contributing to improved outcome of patient safety culture (25, 26). Organizational learning including awareness about patient safety and medical error, and improvements from mistakes still differed among hospitals. The PDCA method was applied in which “Check” and “Action” steps are important to help manage service quality in general and patient safety in particular. However, not all health facilities were properly aware and complied with this method (27). Hospitals should strengthen patient safety training courses and establish a supportive learning environment to improve the knowledge level and skills of health workers, to develop an environment of patient safety culture in the hospital (28).

Our study showed few predictors of overall patient safety: pharmacist, nurse/technologist, and the age of from 50 to 60. This result was in contrast to some studies’ results in which nurses tend to provide a more positive assessment of patient safety culture than doctors and some other groups (21–23). Nurses could make a good cooperative relationships and build trust with other health workers to improve service quality and patient safety (29). Researchers will need to conduct more studies to improve their understanding about the role of nurses in patient safety culture, but this study may suggest solutions to leverage the role of nurses to improve patient safety culture in hospitals. In our study, the age group of 50–60 years was a predictor in Hos2. This finding was in contrast to some studies suggesting that younger age groups had a higher culture of patient safety (30). Therefore, further studies should consider verifying this finding.

### Study limitations

Our research might have faced bias due to the sensitivity of the health worker’s point of view. The accuracy of the study depended strongly on the psychological status and assessment of the study participants, especially contents which were related to the supportive management of leaders. Therefore, health workers could have been dishonest leading to providing inaccurate information. To overcome these issues, we conducted interviews in private places and used a self-report questionnaire to help subjects feel more comfortable answering questions.

## Conclusion

Measuring health staff’s perception of patient safety within healthcare organizations is crucial to identify the strengths and challenges encountered by the organizations; in turn, it helps eliminate medical errors and enhances the health service quality delivered. This study reported that communication openness and organization learning were two aspects that need to be improved as they were not only strongly related to patient safety culture but also to knowledge exchange among health staff. Excitingly, this study revealed the role of nurses as a predictor of patient safety culture among three hospitals. Further investigations are required to produce a comprehensive picture of the role of nurses in improving health personnel relationships, service quality and patient safety culture.

## Data availability statement

The original contributions presented in the study are included in the article/supplementary material, further inquiries can be directed to the corresponding author.

## Ethics statement

The study protocol was approved by the scientific panel from the School of Preventive Medicine and Public Health, Hanoi Medical University in Vietnam. Participation of all respondents was anonymous and voluntary. They were informed about research content and objectives as well as how the interview data would be documented and reported and that their confidentiality would be respected. Participants provided verbal informed consent and could withdraw at any time.

## Author contributions

NTHT, PTH, NTTH, DNTT, and BTMA developed the structure and draft of the manuscript, PHG analyzed data and all authors contributed to the study conception, design and revising of the article, and approved the final version of the manuscript.
